# Real-time measurement of metals in submicron aerosols with particle-into-liquid sampler combined with micro-discharge optical emission spectroscopy

**DOI:** 10.1007/s10661-024-13298-3

**Published:** 2024-10-30

**Authors:** Sudatta Das, Kimmo Teinilä, Hilkka Timonen, Erkki Ikonen, Toni Laurila

**Affiliations:** 1 Metrology Research Institute, Aalto University, Maarintie 8, 02150 Espoo, Finland; 2 Finnish Meteorological Institute, Erik Palménin Aukio 1, 00560 Helsinki, Finland; 3 VTT MIKES, VTT Technical Research Centre of Finland Ltd, Tekniikantie 1, 02150 Espoo, Finland; 4 Customer Application Center, Sensmet Ltd, Otakaari 7,02150Espoo Espoo, Finland

**Keywords:** Trace metals in air, Particle-into-liquid sampler, Micro-plasma emission spectroscopy, Sensitive on site monitoring

## Abstract

The paper presents a novel technique for quantifying trace metals in aerosol samples in real time. Airborne metals were continuously collected for one week near the Baltic Sea in Finland using a particle-into-liquid sampler (PILS). The collected liquid samples were analyzed for metals using micro-discharge optical emission spectroscopy (µDOES). The micro-discharge analyzer is designed to perform real-time, on-site measurements of metal concentrations in aqueous solutions. Currently, µDOES can provide online measurements of 30 metals, with typical detection limits from 0.01 µg/m^3^ to 0.06 µg/m^3^ with a long-term repeatability less than 5%. The novelty of this analyzer lies in its compact design, rapid detection capabilities, and ease of operation and maintenance. Several metals, including potassium (K), calcium (Ca), sodium (Na), aluminum (Al), magnesium (Mg), and copper (Cu), were measured in the aerosol samples collected using PILS. The results indicate that this approach has significant potential for future automated online monitoring of airborne metal concentrations, facilitating investigations into their sources and daily variations. The development of real-time technologies for rapid, online, and accurate atmospheric aerosol measurements is essential for advancing climate change research. Such advancements allowing for continuous real-time data enhance our understanding of aerosol dynamics, improve source identification, and inform public health and environmental policies, ultimately contributing to more effective climate change monitoring and mitigation.

**Supplementary Information:**

The online version contains supplementary material available at 10.1007/s10661-024-13298-3.

## Introduction

Trace metals are essential for human health, but excessive exposure can be harmful. Airborne trace metals are significant contributors to adverse health effects, originating from both biogenic and anthropogenic sources (Paithankar et al., 2021). The major biogenic sources (Garrett, 2000) include volcanic eruption, weathering of rocks, forest fires, and soil erosions, while primary anthropogenic sources (Popescu & Ionel, 2010) include e.g. traffic, biomass combustion, mining, smelting, coal-burning powerplants, uncontrolled agricultural practices, and municipal waste combustion. Atmospheric trace metals and other airborne pollutants are transported in the atmosphere as particulate matter (PM) (Vithanage et al., 2022). PM moves with airmasses and eventually deposits into various ecosystems, such as soil, plants and water, through dry and wet deposition (He et al., 2023). Dry deposition (Wesely & Hicks, 2000) involves transferring of airborne particles to the Earth’s surface, while wet deposition refers to rain or snow scavenging (Cheng et al., 2021) also responsible for removing airborne particles.

Humans can be exposed to metals primarily through inhalation and dermal contact, with air serving as the main medium (Geiger & Cooper, 2010). Hence the analysis of atmospheric aerosols is crucial for understanding exposure pathways and identifying sources of pollution. Trace metals such as sodium (Na), potassium (K), Copper (Cu), magnesium (Mg), aluminum (Al) and others serve as indicators or tracers for various pollution sources due to their distinct characteristics and associations with natural or anthropogenic activities. This information is integral to public health assessments and plays an important role in informing regulatory authorities and environmental management strategies.

Traditional methodologies for analyzing trace metals in atmospheric aerosol often employ techniques such as ion chromatography (IC) or gas chromatography and liquid chromatography coupled with mass spectrometry (GC–MS or LC–MS), as well as inductively coupled plasma mass spectrometry (ICP-MS) and laser-induced breakdown spectroscopy (LIBS). Although these methods are effective in providing detailed insights into trace metal composition, they present several limitations that hinder their suitability for rapid and straightforward on-site measurements. One significant drawback of traditional methods like IC, GC–MS, and LC–MS is the extensive sample preparation required, which can be both time-consuming and labor-intensive. These techniques (Bhowmik et al., 2022; Santos & Galceran, 2002; Steiner et al., 2021) also exhibit limited capabilities for elemental detection and are vulnerable to matrix effects, which can compromise the accuracy of the results. On the other hand, ICP-MS has been successfully used to analyze a wide range of elements in atmospheric aerosols, including trace metals such as potassium (K), sodium (Na), calcium (Ca), aluminum (Al), magnesium (Mg), and copper (Cu), along with various heavy metals in both urban and rural environments (Toscano et al., 2009). However, ICP-MS necessitates expensive instrumentation, a supply of high-purity argon carrier gas, and complex labor-intensive analytical procedures. LIBS (Heikkilä et al., 2022) offers a promising alternative for airborne metal analysis in real time. LIBS features simpler sample preparation than ICP-MS, compact instrumentation, and portability. This method utilizes a high-energy laser pulse directed at the sample, creating a plasma that vaporizes a small amount of material. As the plasma cools, it emits light characteristic of the sample's elemental composition, enabling instantaneous analysis. However, compromised sensitivity of LIBS (Khan et al., 2022) can be a challenging issue in measuring low concentrations of trace metals in atmospheric aerosols. Hence, novel sensitive, fast, compact, and low-cost instruments are needed to analyze airborne metals.

With this contribution, we showcase an online real-time analyzer based on micro-discharge spectroscopy to measure the metal composition in liquid aerosol samples collected using a particle-into-liquid sampler (PILS). The online metal analyzer utilizes micro-discharge optical emission spectroscopy (µDOES®), a technology developed by Sensmet Ltd. in Finland. This innovative tool can simultaneously detect multiple dissolved metals in aqueous solutions at high sensitivity, with metal detection limits down to sub-microgram per liter (sub-µg/L). Its excellent repeatability and ability to perform continuous automated monitoring of multiple metals make it a versatile choice for a wide range of applications. Furthermore, the analyzer’s compact design and straightforward use without carrier gases enable 24/7 operation without the need for personnel, allowing for on-site and real-time measurements.

## Materials and methods

### Study location

The measurement location was at Aalto university, Espoo (Maarintie 8 building,60.18º N,24.82º E), Finland. The building is located at university campus area, around 700 m from the Baltic Sea. The building consists of five auditoriums, library and a restaurant also near to an industrial park. The samples were collected on the roof of the building’s third floor.

### Instrumentation and sampling

PILS is a commercially available specialized device used for aerosol collection (Weber et al., 2001). Traditionally, airborne particles have been collected using membrane filters, polycarbonate filters, and quartz filters (Soo et al., 2016). However, these sampling methods can encounter issues such as sampling artifacts, particle loss, and potential biases during chemical analysis (Orsini et al., 2003). In contrast, PILS is preferred over traditional filters because it collects samples directly into liquid form, making them more suitable for analysis. It also allows for continuous sampling, which helps minimize problems like evaporation and chemical reactions. Furthermore, PILS does not suffer from particle loading, ensuring more accurate and reliable results.

In our setup, outside air was drawn at a constant rate into the PILS using a 2-m-long copper tube with an inner diameter of 10 mm. Within the PILS the air sample gets mixed with steam at around 100º C generated by a small turbulent water flow. The steam cools down upon mixing it with the sample air flow to create a condition where the chamber is supersaturated with water vapor. In the supersaturated condition, the aerosol particles grow into droplets (Watson, 2016) and are collected on the impactor surface flushed with a constant flow of ultrapure water. The flushing process, along with water vapor condensation, leads to dilution. The dilution effect was quantified by spiking the ultrapure water used for PILS with a known concentration of non-interfering metal (Sorooshian et al., 2006). In our experiment, the ultrapure water was spiked with 100 µg/L of barium (Ba) during the collection. The diluted barium concentration exiting the impactor was then measured to account for the added water. The ambient concentration was quantified by the following equation (Orsini et al., 2003):

1 $${C}_{a}={C}_{L}{q}_{in}R/Q$$

where *C*_a_ is the ambient aerosol concentration (µg/m^3^), *C*_L_ is the concentration of the metals in the collected liquid sample (µg/L), *q*_in_ is the flow of the spiked transport liquid entering the top of the impactor (typical flow rate used here was 0.06 to 0.14 ml/min), *R* is the ratio of the spiked barium concentration entering the impactor to the concentration exiting the impactor (ranging here from 0.99 to 1.3), and *Q* is the volumetric flow rate of sample air entering the PILS (here a 16.3 L min^−1^ flow rate was applied).

The metal analysis was performed using a µDOES analyzer (Das et al., 2023; Blomberg et al., 2012), which employs micro discharge in water to detect and quantify metallic elements in samples. The analyzer applies high-voltage electric pulses with a repetition rate ranging from 500 to 1000 Hz between two tungsten electrodes. These pulses create micro-discharges inside the aqueous sample, which is placed in a quartz measurement cell at atmospheric pressure during the measurement. The aqueous samples are automatically transported from the sample bottle to the analyzer's measurement cell using analyzer’s peristaltic pumps. This eliminates the need for a nebulizer and other high-maintenance, fragile components commonly used in ICP-MS/OES instruments. During the micro-discharge process, a microscopic volume of the sample liquid undergoes flash heating, reaching a temperature of approximately 10,000 K. In this intense heat, the molecular species present in the sample are dissociated into atoms, which are then excited to their respective higher electronic states. As the excited atoms return to their ground state, they emit light at specific wavelengths corresponding to their characteristic energies. The emitted light is captured by an array-based spectrometer, which allows for the quantitative analysis of the metals present in the aqueous sample. The µDOES analyzer covers the spectral range between 200 and 850 nm, ensuring a comprehensive detection of various metals. The identification of emission lines is based on the NIST database (Ralchenko, 2005), which provides a reliable reference for metals identification.

The sample collection process began with a continuous reference blank sample collection in late July, followed by atmospheric aerosol samples collected continuously outdoors for eight days from 26th of July to 2nd of August 2023. The reference blank samples were obtained by circulating ultrapure water through the PILS system without airflow. Ideally, the metal concentrations in the reference samples are much smaller than those observed in the atmospheric aerosol samples. The effect of these residual metals was minimized here by thorough flushing of the PILS system and the associated sampling lines with 1 L of ultrapure water for 3 to 4 h before the measurements. Finally, the liquid samples were stored in Teflon (PTFE) bottles and preserved in a refrigerator for metal measurements using the µDOES analyzer. Each sample Teflon bottle, containing about 100 mL of liquid, was collected from outside air starting at noon for a duration of approximately 20 h. The liquid samples were clear and transparent and measured without preparation, while a magnetic stirrer was employed to ensure uniform distribution of trace elements during measurement.

## Results

### Analysis of samples

Traces of K, Ca, Na, Mg, Cu and Al were detected in the liquid samples containing the collected aerosols. Ba was also analyzed, as it was used as a spiking metal to account for the dilution effect. The emission spectrum of each liquid sample was recorded three times (*n* = 3) and averaged for analysis.

**Fig. 1:**
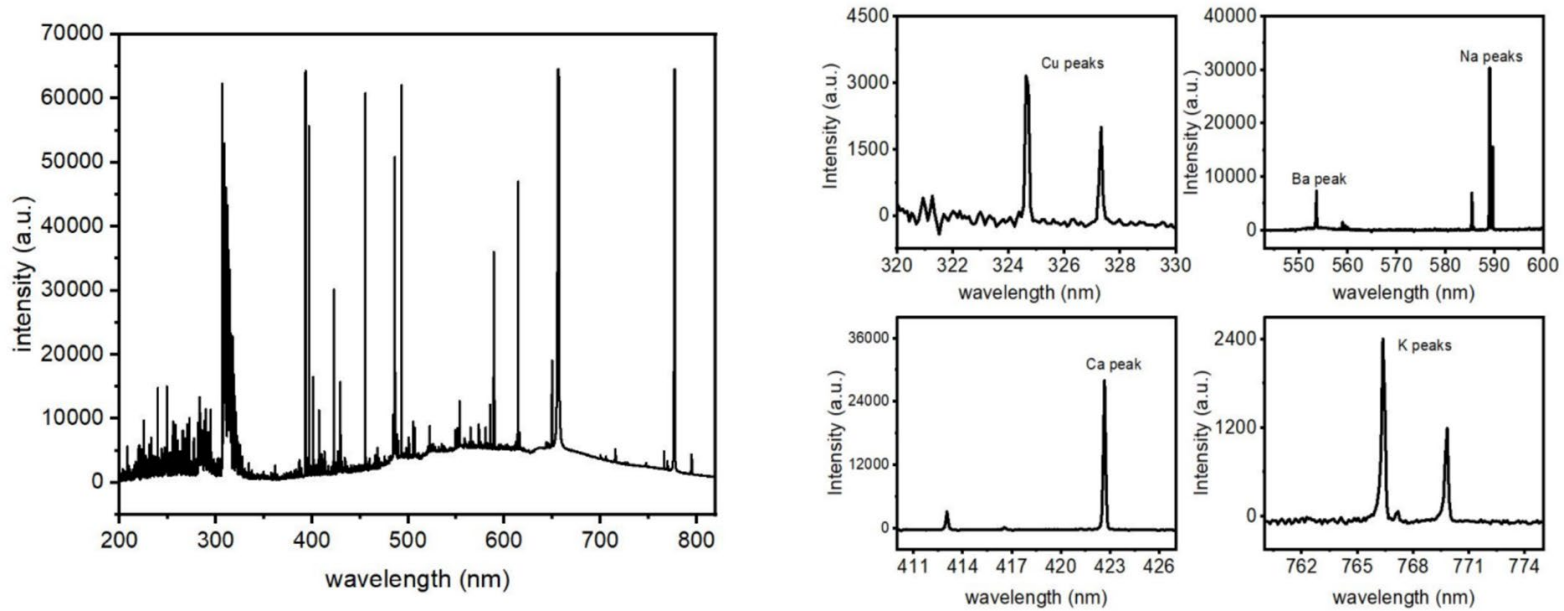
left) Raw 200–800 nm emission spectrum from an aerosol containing liquid sample. right) emission spectra of Cu, Na, Ba, Ca and K after background removal using calibration models

Quantitative metal concentrations were determined using calibration models developed for the µDOES analyzer. Figure 1 illustrates an emission spectrum of an aerosol sample, with the left side displaying the raw emission spectrum from a liquid aerosol sample and the right side showing the background-subtracted spectra, highlighting the distinct metal peaks. The strong emission lines in Fig. 1 are identified as follows: Cu I peaks at 324.7 nm and 327.3 nm, Ca I at 422.64 nm, Na I at 589 nm and 589.61 nm, Ba at 553.54 nm, and K I peaks at 766.46 nm and 769.92 nm.

The mathematical calibration model reconstructs the measured emission spectrum by incorporating the spectra of pure metal solutions from the library, a background matrix spectrum, and any species that spectrally interfere. The pure element library spectra are based on the measurement of CRM (certified reference material) standards. Once the calibration model is prepared, it is then fitted to the measured emission spectra using the least squares method. This process determines the unknown concentration of the samples being studied. The approach facilitated the determination of the unknown concentrations of trace metals in aerosol samples. At this stage, we subtract the concentrations of the reference blank samples from each outdoor sample to eliminate the influence of any residual metals present in the PILS system.

### Analytical performance of µDOES

The instrument was calibrated for K, Ca, Na, Al, Mg, Cu, and Ba concentrations from 25 µg/L to 200 µg/L. The detection limits for the above metals are established based on signal-to-noise ratio (SNR) (Shrivastava & Gupta, 2011). A signal of three times the standard deviation of background noise was considered as the threshold for detecting the signal. Hence, it is important to calculate the background noise for each metal spectrum. To accurately determine the background noise level, ultrapure deionized water was utilized to prepare blank standards. The analyzer software employs the calibration model fitted to these blank standards for each metal, subsequently computing the standard deviation from ten repeated blank measurements. The mathematical representation of the calibration curves using linear equation and other analytical figures of merit are summarized in Table 1. The linear equation models the relationship between the known standard concentrations (*x*) and the measured analyte concentrations (*y*) obtained through the calibration process. The equation takes the general form: *y* = *mx* + *b*, where *m* is the slope, *b* is the intercept, and the method demonstrates a robust linear response (*R*^2^ = 0.990 or better) with good repeatability (measured in terms of relative standard deviation) over 10 sample measurements, as shown in Table 1.

**Table 1:** Analytical performance characteristics of the metals monitored using the µDOES analyzer

Element	Emission wavelength (nm)	Linear equation	Limit of Detection (in µg/L)	Limit of Detection (in µg/m^3^)^*^	Linearity (adjusted *R*^2^ values)	Relative standard deviation (%)
K	766.46 and 769.92	1.02x-4.05	2.1	0.014	0.990	4.3
Ca	422.64	0.987x + 3.94	1.8	0.012	0.997	3.6
Na	589 and 589.61	1.013x + 0.016	0.2	0.001	0.998	1.5
Al	394.38 and 396.12	1.0285x-1.0551	1.3	0.009	1.000	1.9
Mg	517.30	0.976x + 2.582	7.8	0.053	0.999	4.5
Ba	553.54	0.899x + 10.07	3.4	0.023	0.999	4.3
Cu	324.702 and 327.314	1.15x + 5.314	1.3	0.009	0.999	2.7

*The ambient concentration is calculated based on airflow rate, spiked liquid flow rate and dilution ratio

### Daily variation of metal concentration

**Fig. 2:**
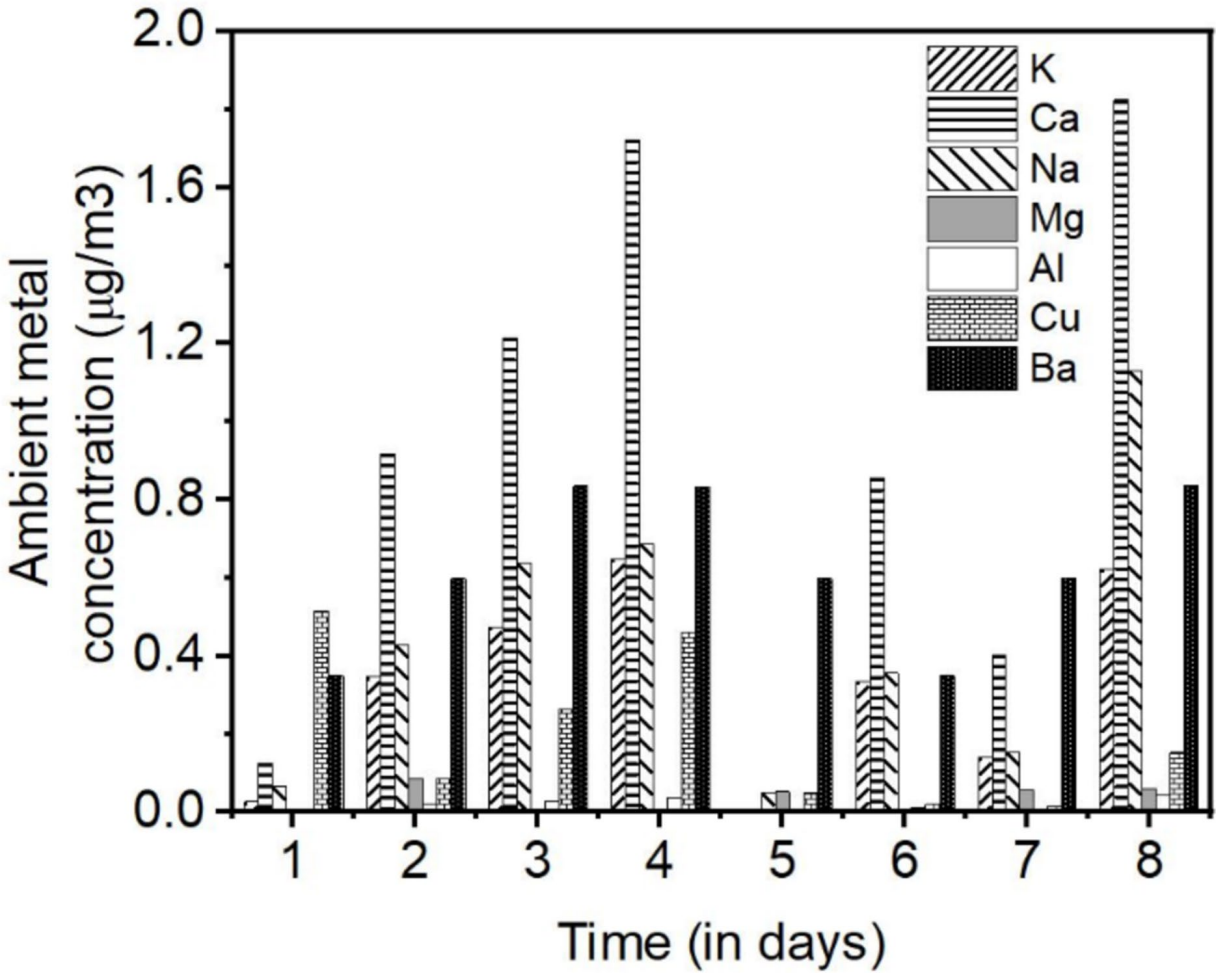
Concentration variation of different metals in ambient outdoor aerosol samples collected over 8 days. There is no Ba present in the ambient aerosol. The displayed Ba concentration is a result of remnant Ba from the spiking used during the process in the setup

Figure 2 illustrates the daily concentration variation of metals observed for the aerosol samples collected over days 1 to 8. Metal concentrations below the detection limit are not represented in the diagram. To track the dilution ratio for each sample, barium concentrations were also measured and are depicted in Fig. 2. The variation (minimum to maximum concentration) of ambient aerosol concentration is calculated using Eq. 1 and is presented in Table 2.

**Table 2:** Ambient metal concentration in aerosol samples observed over 8 days

Metal in aerosol	Ambient metal concentration range (µg/m^3^)	The multi-annual metal concentration range (µg/m^3^) in Helsinki, Finland, by Ioannidou et al., 2023
K	0.03 to 0.65	< 0.01 to 1.12
Ca	0.13 to 1.72	0.01 to 1.76
Na	0.05 to 0.64	0.07 to 1.37
Al	0.02 to 0.04	< 0.01 to 1.04
Mg	0.06 to 0.09	NA
Cu	0.02 to 0.52	< 0.01 to 0.44

These measured values were compared with a long-term analysis of urban air in Helsinki, Finland (Ioannidou et al., 2023) covering a study period from 1962 to 2005. The results presented in Table 2 demonstrate the very good general agreement between the current observations using the novel online method and the long-term reference values. Overall, the metal concentrations in the aerosol samples varied from low levels of less than 0.2 µg/m^3^ to significantly higher concentrations of up to 2 µg/m^3^. The highest ambient concentrations were observed in the following order: Ca > K > Na > Cu > Mg > Al. The greatest variation in concentration was noted for Ca, followed by K, Na and Cu. It is important to highlight that alkali and alkaline earth metals (Na, Ca, K) and Cu were present in all analyzed aerosol samples, while traces of Al and Mg were detected in only some samples.

Although analyzing the sources of metals in atmospheric aerosols is beyond the scope of this article, some insights can be glanced from Fig. 2. For instance, there is a significant and sudden drop in metal concentration, attributed to the washout process caused by rainfall during the fifth day. To understand the effect of marine or non-marine sources on metal concentrations, we calculated the mass concentration (µg/m^3^) of sea salt and non-sea salt fractions for K and Ca using the equations in reference (Brewer, 1975). The results indicate that more than 90% of Ca and K originate from non-marine sources, suggesting that soil or biomass burning may be a significant contributor. Also, based on the site’s location, it can be inferred that the primary source of Na is the sea nearby whereas K and crustal metals such as Ca, Mg, and Al in aerosols usually originate from wind-blown soil dust (Chesselet et al., 1972; Vaalgamaa & Conley, 2008). Furthermore, diesel engines and industrial emissions have been identified as major sources of Cu in the atmosphere (Lee Jr and Lehmden, 1973; Pulles et al., 2012).

Finally, the uncertainty in ambient aerosol mass measurement comprises three key components: the uncertainty of analyzer calibration (*u*(*c*)), the uncertainty associated with the airflow rate (*u*(*a*)) and the uncertainty of the liquid flow rate (*u*(*l*)) of PILS. Our previous study (Das et al., 2023) indicated that the relative standard uncertainty *u*(*c*) for most metals measured using the analyzer is 4.2%. Meanwhile, *u*(*a*) was calculated based on the standard deviation of daily airflow rates during measurements, yielding a value of 4.3%. Additionally, *u*(*l*) is estimated to be 11.4%, based on standard deviation of daily flow rate at which the liquid enters the PILS system. Consequently, the combined relative standard uncertainty can be expressed as follows: $\sqrt{{u(c)}^2+{u(a)}^2+{u(l)}^2}=12.6\%$

## Conclusion

Understanding the metal content in atmospheric aerosols is crucial for assessing their environmental impact. However, there is currently a significant gap in affordable instrumentation capable of real-time elemental analysis with low enough detection limits, which is essential for quantifying concentrations in field conditions. In this context, we present a promising new method that combines a particle-into-liquid sampler (PILS) with a micro-discharge-based liquid analyzer (µDOES). This innovative approach shows great potential for on-site aerosol analysis for metals. While PILS provides reliable results for water-soluble species, it is not without limitations. One notable concern is the potential for cross-contamination from artifacts, which necessitates a routine cleaning of the sample lines and body on a weekly basis. In contrast, µDOES offers a significant advantage over traditional analyzers such as IC, LC/GC–MS and ICP-MS. It requires less frequent maintenance, with a typical interval of up to three months, rather than the daily to weekly upkeep needed for many existing methods. In addition, research is ongoing to enhance the analyzer's speed and to achieve higher sensitivity. The µDOES analyzer has currently been demonstrated to be capable of measuring 30 metals in real time so there is good future potential to expand the metals coverage beyond the six metals demonstrated in this study. The benefits of the combined PILS-µDOES method include sensitivity and flexibility, allowing users to choose to collect and analyze samples in real-time or conduct offline analysis at a later time. This new approach represents a significant advancement in the field of aerosol analysis, paving the way for more effective environmental monitoring.

## Supplementary Information

Below is the link to the electronic supplementary material.

ESM 1 (DOCX 1.05 MB)

## Data Availability

The data that support the findings of this study are available upon reasonable request from the corresponding author.
