# Supplementary material for: Real-time measurement of metals in submicron aerosols with particle-into-liquid sampler combined with micro-discharge optical emission spectroscopy
Source: Environ Monit Assess. 2024 Oct 30;196(11):1128. doi: 10.1007/s10661-024-13298-3 (PMC11525318; doi:10.1007/s10661-024-13298-3)

Figure : Up) Schematic diagram of PILS used for aerosol sample collection. Down) A schematic diagram of µDOES shows the micro-discharge created inside the aqueous sample between the two electrodes.


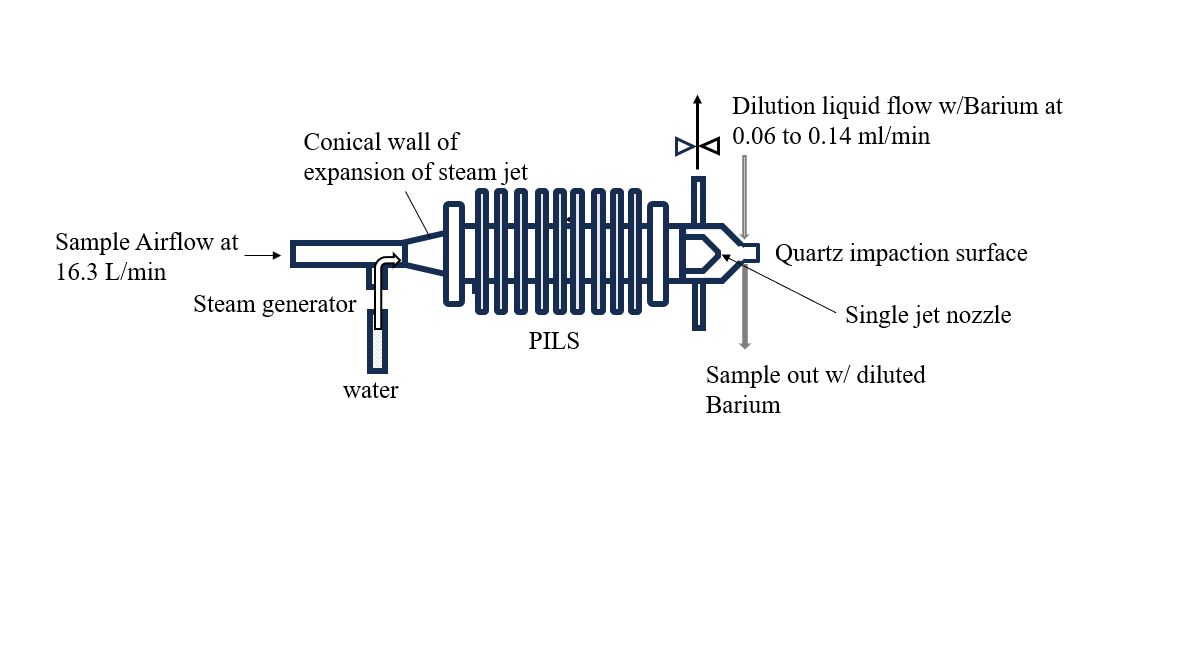

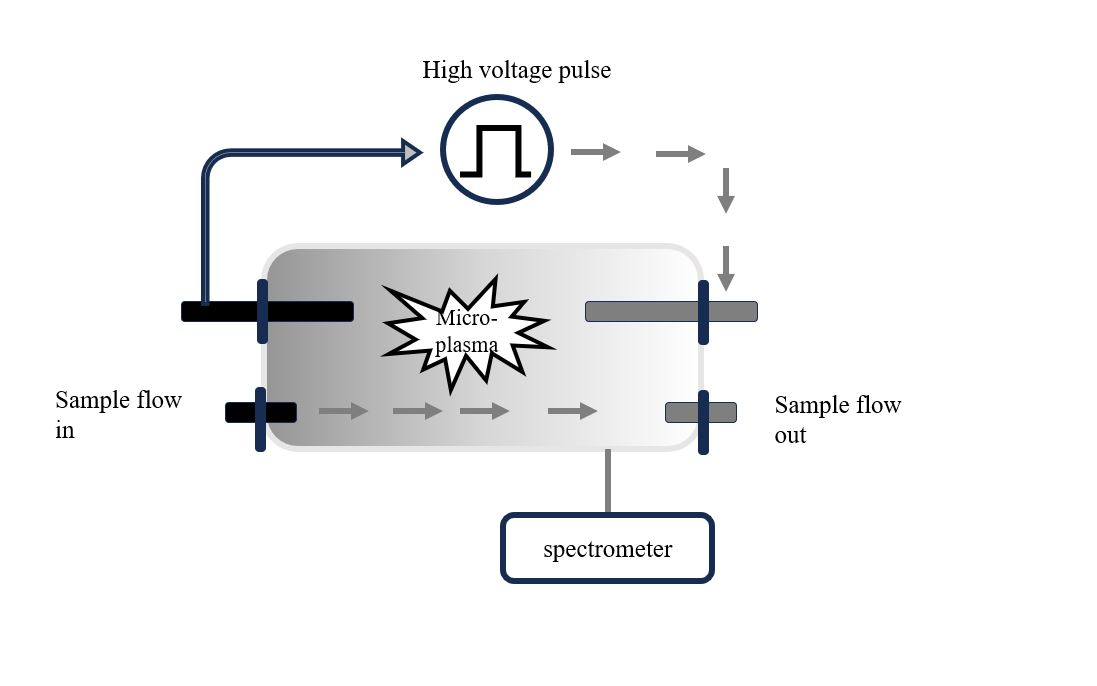

Supplement: Supplementary file 1 — (DOCX 1.05 MB) [file 10661_2024_13298_MOESM1_ESM.docx]
